# Impairments in Fear Extinction Memory and Basolateral Amygdala Plasticity in the TgF344-AD Rat Model of Alzheimer’s Disease Are Distinct from Nonpathological Aging

**DOI:** 10.1523/ENEURO.0181-22.2022

**Published:** 2022-06-23

**Authors:** Caesar M. Hernandez, Nateka L. Jackson, Abbi R. Hernandez, Lori L. McMahon

**Affiliations:** 1 Department of Cellular, Developmental, and Integrative Biology, The University of Alabama at Birmingham, Birmingham, Alabama 35294-0006; 2 Evelyn F. McKnight Brain Institute, The University of Alabama at Birmingham, Birmingham, Alabama 35294-2182; 3 Department of Medicine, Division of Gerontology, Geriatrics, and Palliative Care, The University of Alabama at Birmingham, Birmingham, Alabama 35294; 4 Nathan Shock Center of Excellence in the Basic Biology of Aging, The University of Alabama at Birmingham, Birmingham, Alabama 35294; 5 Integrative Center for Aging Research, The University of Alabama at Birmingham, Birmingham, Alabama 35294

**Keywords:** aging, Alzheimer’s disease, basolateral amygdala, electrophysiology, fear extinction, neurobiology

## Abstract

Fear-based disorders such as post-traumatic stress disorder (PTSD) steepen age-related cognitive decline and double the risk for developing Alzheimer’s disease (AD). Because of the seemingly hyperactive properties of fear memories, PTSD symptoms can worsen with age. Perturbations in the synaptic circuitry supporting fear memory extinction are key neural substrates of PTSD. The basolateral amygdala (BLA) is a medial temporal lobe structure that is critical in the encoding, consolidation, and retrieval of fear memories. As little is known about fear extinction memory and BLA synaptic dysfunction within the context of aging and AD, the goal of this study was to investigate how fear extinction memory deficits and basal amygdaloid nucleus (BA) synaptic dysfunction differentially associate in nonpathologic aging and AD in the TgF344AD (TgAD) rat model of AD. Young, middle-aged, and older-aged WT and TgAD rats were trained on a delay fear conditioning and extinction procedure before *ex vivo* extracellular field potential recording experiments in the BA. Relative to young WT rats, long-term extinction memory was impaired, and in general, was associated with a hyperexcitable BA and impaired LTP in TgAD rats at all ages. In contrast, long-term extinction memory was impaired in aged WT rats and was associated with impaired LTP but not BA hyperexcitability. Interestingly, the middle-aged TgAD rats showed intact short-term extinction and BA LTP, which is suggestive of a compensatory mechanism, whereas differential neural recruitment in older-aged WT rats may have facilitated short-term extinction. As such, associations between fear extinction memory and amygdala deficits in nonpathologic aging and AD are dissociable.

## Significance Statement

Adults with fear-based disorders like post-traumatic stress disorder are at an increased risk for developing age-related cognitive decline and Alzheimer’s disease (AD). Moreover, negative emotional affect is an early marker of AD. The link between fear-based disorders and AD creates a disadvantage for achieving positive outcomes later in life. Central to the circuitry underlying fear disorders are medial temporal lobe structures like the basal amygdaloid nucleus (BA). However, the role of the BA in fear-based disorders exacerbated by aging and AD is not well understood. Using the TgF344AD rat model of AD, we investigated how fear extinction memory impairments and BA synaptic function are impacted by aging and AD, and whether these processes differentially associate in nonpathologic aging and AD.

## Introduction

Nonpathologic aging is accompanied by impaired executive functions (Buckner, 2004) and a greater prevalence of fear-based neuropsychiatric disorders such as post-traumatic stress disorder (PTSD), general anxiety disorder, and panic disorder (Floyd et al., 2002; Beaudreau and O’Hara, 2008). Because of the persistent and hyperactive properties of fear memories, symptoms of fear-based disorders can worsen with age (Beaudreau and O’Hara, 2008). Unfortunately, younger individuals who managed to recover from PTSD often have a recurrence in old age (Floyd et al., 2002). Critically, PTSD significantly steepens age-related cognitive decline (Yehuda et al., 2005; Green et al., 2016), associates with greater tau accumulation (Mohamed et al., 2019), and doubles the risk of developing Alzheimer’s disease (AD) and other dementia in older individuals (Qureshi et al., 2010; Yaffe et al., 2010).

Though nonpathologic and pathologic aging are cognitively and biologically dissociable, aging is the greatest risk factor for the development of neurodegenerative diseases such as AD (Swerdlow, 2007; see https://www.nia.nih.gov/health/what-causes-alzheimers-disease). Indeed, cognitive and emotional impairments worsened by neuropsychiatric disorders like PTSD in old age may be indicative of an irreversible trajectory toward developing AD. Consistent with this are the findings that individuals with AD have a greater prevalence of fear-based disorders (Burke et al., 2018). Moreover, aversive emotional memories, such as those associated with fear-based neuropsychiatric disorders may be an early marker of AD and dementia (Neary et al., 1998; McKhann et al., 2001; Hoefer et al., 2008), even before other measurable cognitive deficits.

While the focus of many cognitive aging and AD studies has been on the prefrontal cortex (PFC) and hippocampus (HPC), a unifying thread linking PTSD, cognitive aging, and AD is aberrant activity in the amygdala (Shin et al., 2006; Wright et al., 2007). Topographical studies have established the basal amygdaloid nucleus (BA) of the basolateral amygdala (BLA) complex receives abundant inputs from the PFC and HPC (McDonald et al., 1996; McDonald and Mott, 2017). The BLA provides emotional valence (positive or negative) to memory (Garavan et al., 2001; Belova et al., 2008; Beyeler et al., 2016) and serves as an integrative hub in the underlying processes associated with fear memory encoding, consolidation, and retrieval (Maren, 2001; Kochli et al., 2015). Specifically, synaptic communication between the BLA and PFC supports the extinction of fear memories (Peters et al., 2009), whereas communication between BLA and HPC supports fear acquisition and the modulation of fear extinction (Maren and Quirk, 2004; Sierra-Mercado et al., 2011). Critically, the inability to extinguish hyperactive fear memories is a core component of PTSD driven by a hyperexcitable BLA that may be exacerbated by aging and AD (Shin et al., 2006; Wright et al., 2007).

The neurobiology of cognitive aging is loyally recapitulated in rat (Burke and Foster, 2019), and the transgenic Fisher 344 AD (TgAD) rat has been well characterized as a model of AD that comprehensively recapitulates disease progression. Specifically, the TgAD rat model develops an age-dependent increase in cortical and hippocampal amyloid pathology that is present as early as 6 months of age (Cohen et al., 2013). Notably, hyperphosphorylated tau forms endogenously by 6 months of age in locus coeruleus and cingulate cortex, and by 16 months of age it is found in hippocampus (Cohen et al., 2013; Rorabaugh et al., 2017). There are also increases in markers of neuroinflammation by 6 months in the cortex and signs of neurodegeneration by 16 months of age (Cohen et al., 2013). Importantly, the TgAD rat model also recapitulates cognitive decline across the life span, and early-life anxiety (Cohen et al., 2013; Pentkowski et al., 2018). Given the recent link between fear-based disorders, cognitive aging, and Alzheimer’s disease (Burke et al., 2018), the current study leveraged this rat model of AD to collectively assess the effects of nonpathologic aging and AD on fear extinction memory and BA synaptic function in addition to the relationship between these factors. Indeed, defining the synaptic mechanisms linking these factors is foundational for the development of interventional strategies to remediate poor outcomes in aging and neurodegeneration. As a major step toward this goal, young adult (YA), middle-aged (MA), and older-aged (OA) WT and TgAD rats received fear conditioning and extinction behavior, followed by extracellular field recording in slices containing the BA. We then used a principal component analysis (PCA) to determine whether associations between fear extinction and BA function differed by age and AD. In general, our results demonstrate aging in the presence or absence of AD result in unique fear extinction impairments and BA synaptic deficits.

## Materials and Methods

### Subjects

A total of 101 (Table 1, sample sizes) wild-type (WT) and TgF344-AD (TgAD) rats were used for behavioral and *ex vivo* brain slice electrophysiological experiments. As previously described (Smith and McMahon, 2018; Goodman et al., 2021), TgAD rats harboring the human Swedish mutation amyloid precursor protein (APP^swe^) and the presenilin-1 exon 9 deletion mutant (PS1^ΔE9^) were bred with WT F344 females [Envigo (previously Harlan Laboratories)] at the University of Alabama at Birmingham. All breeding and experimental procedures were approved by the University of Alabama at Birmingham Institutional Animal Care and Use Committee and followed guidelines set by the National Institutes of Health. The original breeding pair was obtained from University of Southern California (Los Angeles, CA; Cohen et al., 2013). Of the 101 rats, 19 24-month-old WT rats were obtained from the National Institute on Aging aged rodent colony that is maintained by Charles River Laboratories. Rats were maintained under standard animal care facility conditions with food (catalog #Harlan 2916, Teklad Diets) and water *ad libitum* and a 12 h reverse light/dark cycle (lights Off at 7:00 A.M.) at 22°C and 50% humidity. Rats were housed in standard rat cages (height, 7 inches; floor area, 144 square inches) in same-sex groups of four or fewer at weights of ∼300 g or two per cage at weights ≥400 g. Rats were aged from birth to experimental age groups categorized as YA (age range, 5.67–7.59 months; average age, 6.39 months), MA (age range, 12.17–14.16 months; average age, 13.36 months), and OA (age range, 22.65–25.13 months; average age, 24.53 months) rats. While all rats were included in behavioral experiments, only a subset of these rats were assigned to electrophysiological experiments. A maximum of 2 weeks before contextual fear conditioning, all rats were single housed under the same conditions to avoid experimental confounds (e.g., premature contextual exposure) and given environmental enrichment packs. Final group sizes for behavioral and electrophysiological experiments are described in Table 1.

**Table 1:** All group sample sizes for behavioral and electrophysiological experiments

	YA				MA				OA			
	WT		TgAD		WT		TgAD		WT		TgAD	
	M	F	M	F	M	F	M	F	M	F	M	F
Behavior (ns)	8	9	8	7	8	12	11	11	5	16	4	2
Electrophysiology (ns)	5	4	4	4	4	6	6	5	3	6	3	2

A subset of all the rats that received contextual fear conditioning were assigned to electrophysiological experiments, and as such both behavioral and electrophysiological measures were available for each rat. M, Male; F, female.

### Contextual fear conditioning

#### Fear memory acquisition

**Figure 1.**
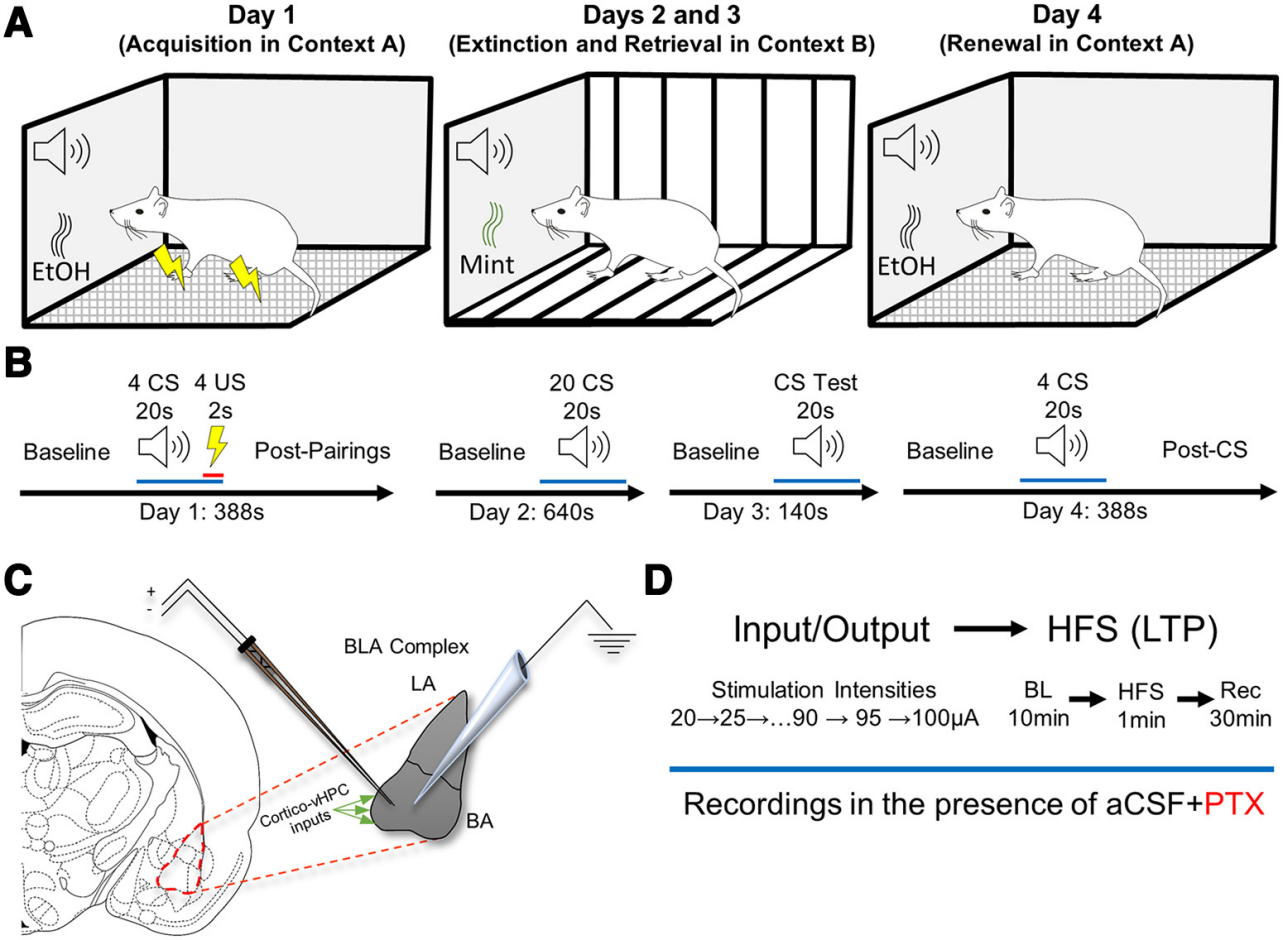
Contextual fear conditioning and electrophysiology experimental paradigm. ***A***, Schematic of day-dependent contextual design. ***B***, Trial schematic. Rats progressed through a 4 d fear-conditioning paradigm in which acquisition in context A took place on day 1, fear extinction in context B took place on day 2, extinction retrieval in context B took place on day 3, and fear renewal in context A took place on day 4. See text in Materials and Methods for more detail. ***C***, In a subset of behaviorally characterized animals, extracellular recordings targeted the basal amygdaloid nucleus of the BLA complex. Topographical studies of the basolateral amygdala describe abundant corticohippocampal inputs to the anterior part, known as the BA, anterior part. To maximize stimulation of these inputs, the stimulating electrode was consistently placed toward the medioventral region of the basolateral amygdaloid nucleus anterior part, and the recording electrode was consistently placed within 1 mm lateral relative to the stimulating electrode. ***D***, Procedure and timeline schematic describing electrophysiological experiments. LA, Lateral Amygdaloid Nucleus; BA, Basal Amygdaloid Nucleus; vHPC, Ventral HPC.

Day 1 consisted of fear memory acquisition in context A (Fig. 1*A*), which consisted of a custom operant conditioning chamber (29.53 × 23.5 × 20.96 cm; Med Associates) composed of featureless walls, a metal grated floor that delivered the shock, and an EtOH scent. Before testing, all rats were habituated in context-specific holding rooms for a minimum of 15 min, and the transportation route between vivarium and holding room was specific to context A. After a 2 min baseline (BL) to determine basal freezing behavior, rats received four trials consisting of a 20 s tone (2000 Hz at 85 dB), serving as the conditioned stimulus (CS), paired with a footshock (1 mA), serving as the unconditioned stimulus (US), during the last 2 s of the tone (CS and US coterminated). Each trial was separated by a 20 s intertrial interval. After the final CS–US pairing, a 2 min postparing epoch was used to determine fear memory acquisition in what became the “unsafe” context (Fig. 1*B*, detailed schematic). As freezing is a response to threat in rodents, fear expression (and by proxy, fear memory) is operationalized as freezing behavior of at least 1 s except for breathing. Freezing activity video was recorded during each session (Video Freeze, Med Associates). Time spent freezing per each epoch [baseline, CS, US, and postpairings (PPs)] was divided by the total time of each epoch to generate a percentage of time (%time) freezing per epoch.

#### Fear memory extinction

Day 2 consisted of fear memory extinction in context B (Fig. 1*A*). Context B consisted of a different operant conditioning chamber from that used in context A and was composed of striped walls, striped plastic flooring that covered the metal grating, and a peppermint scent. Additionally, the holding room during the 15 min habituation period was context specific, and the transportation route between vivarium and holding room was specific to context B. After a 2 min baseline, rats received 20 trials consisting of the CS (tone) only (i.e., in the absence of the US). Each trial was separated by a 5 s intertrial interval (Fig. 1*B*). As there was no US, context B became the “safe” context.

#### Fear memory extinction retrieval (extinction memory)

Day 3 consisted of fear extinction retrieval test (i.e., recall the CS is no longer threatening) in context B (Fig. 1*A*). After a 2 min baseline, rats received 10 trials consisting of the CS only (Fig. 1*B*). Each trial was separated by a 5 s intertrial interval.

#### Fear memory renewal

Day 4 consisted of fear memory renewal, in which rats were placed back into context A (the unsafe context) to test whether their fear response was renewed (Fig. 1*A*). After a 2 min baseline, rats received four trials consisting of the CS only. Each trial was separated by a 20 s intertrial interval. After the final CS, there was a 2 min post-CS epoch used to determine fear memory renewal to context A (Fig. 1*B*).

### Extracellular field excitatory postsynaptic potential recordings in the BLA

#### BLA slice preparation.

Brain slices containing BLA were prepared from rats a minimum of 2 weeks after contextual fear conditioning. Rats were anesthetized via deep isoflurane inhalation and rapidly decapitated, and brains were rapidly extracted. Coronal slices (400 μm) containing BLA were prepared using a vibratome (model VT1000P, Leica). As per Goodman et al. (2021), slices were made in low Na^+^, sucrose-substituted ice-cold artificial CSF (aCSF) containing the following (in mm): NaCl (85), KCl (2.5), MgSO_4_ (4), CaCl_2_ (0.5), NaH_2_PO_4_ (1.25), NaHCO_3_ (25), glucose (25), and sucrose (75), saturated with 95% O_2_, 5% CO_2_, at pH 7.4. Slices were held in a water bath at 26°C for 30 min in standard aCSF containing the following (in mM): NaCl (119.0), KCl (2.5), MgSO_4_ (1.3), CaCl_2_ (2.5), NaH_2_PO_4_ (1.0), NaHCO3 (26), and glucose (11), saturated with 95% O_2_, 5% CO_2_, at pH 7.4 before transfer to the submersion chamber for recordings.

#### Stimulus response (input/output)

To determine the strength of basal synaptic transmission, brain slices containing BLA were prepared from rats a minimum of 2 weeks after contextual fear conditioning (Fig. 1*C*). Extracellular field EPSPs (fEPSPs) were recorded from BLA slices in a submersion chamber continuously perfused with warm (27–29°C) aCSF containing the GABA_A_ channel blocker picrotoxin (PTX; 40 μm) to isolate excitatory transmission (Fig. 1*D*). Baseline fEPSPs were generated using a bipolar simulating electrode placed within 1 mm of an aCSF-PTX-filled glass recording electrode and by stimulating the medial portion of the BA (0.1 Hz for 200 μs) to activate cortical and hippocampal inputs. The fEPSP peak amplitude was measured at increasing stimulus intensities in 5 μA steps from 20 to 100 μA. Paired-pulse stimulation (interval, 50 ms) was delivered throughout the experiment to assess facilitation or depression.

#### Long-term potentiation

Immediately following input/output (I/O) experiments (and while maintaining the same aCSF + PTX perfusion; Fig. 1*D*), the simulation intensity was adjusted to generate ∼50% of the maximum peak amplitude response and a 10 min baseline was recorded before delivering high-frequency stimulation (HFS) to induce long-term potentiation (LTP; HFS: four trains of 100 Hz, 500 ms duration, separated by 20 s; Huang and Kandel, 2007; Humeau et al., 2007). The fEPSP peak amplitude was measured, transformed to the percentage of BL (%BL), and plotted over time. The LTP magnitude was measured by comparing peak amplitude at 30 min post-tetanus to baseline.

### Statistical analysis and experimental design

#### General statistical approach

Unless otherwise noted, all statistical analyses were performed using SPPSS28 version 280.0.0(190). In all analyses, the α was set to 0.05, and when Mauchly’s test of sphericity was violated, the Huynh–Feldt *p*-value correction was applied. When there were significant effects, the effect sizes were reported as η_p_^2^ for ANOVAs and Cohen’s *d* for *t* tests. Additionally, observed power for significant effects was reported as 1-β. For brevity, all null effects were reported as consolidated *F*-statistics (or *t*-statistics) and *p*-values, and only the lowest and highest values for each were given. Outliers were identified as rats that expressed preconditioned freezing during the acquisition baseline epoch using the outlier identification analysis in SPSS. In all analyses, genotype and sex were coded as nominal variables, age was coded as an ordinal variable, and any repeated measure was coded as continuous. All figures were generated in GraphPad Prism version 9.3.0(463).

#### Contextual fear conditioning

In general, a hierarchical approach was used in all data analyses. The percentage of time freezing was initially analyzed using a mixed-factor ANOVA (age × genotype × sex × trial) with age (three levels: YA, MA, and OA), genotype (two levels: WT and TgAD), and sex (two levels: male and female) as between-subjects factors, and trial (six levels for acquisition: BL, CSUS1, CSUS2, CSUS3, CSUS4, and PP; four levels for extinction, retrieval, and renewal: fear memory probe trial (Mem), last extinction trial (ExL), first retrieval (Ret), and renewal (Ren); and three levels for context: context A during acquisition (A_1_), context B, and context A during renewal (A_2_)) as the within-subjects factors. While the effects of sex are reported, sex is underpowered in older-aged TgAD rats. Therefore, only the main effects and interactions between age and genotype were followed up with pairwise comparisons using Fisher’s least significant difference. Significant pairwise comparisons were reported as the mean difference followed by *p*-values. A second tier of analysis was used that focused on the effects of genotype within an age group by using a two-factor ANOVA (genotype × trial). Finally, to determine whether individual groups were successful at acquisition, extinction, retrieval, and renewal, paired-samples *t* tests were used for each group separately. For acquisition, paired-samples *t* tests compared BL to PP. For extinction and retrieval, paired-samples *t* tests compared both the Ext trial and the Ret trial to the Mem trial. For renewal, paired-samples *t* tests compared Ren to Ret. To compare the percentage of time freezing during context testing, paired-samples *t* tests were used between A_1_ or A_2_ to context B (after extinction training).

#### Behavioral control measures

To account for the possibility that differences in %time freezing during were confounded by shock perception, motion data were extracted from US epochs, transformed to reflect the relative change in motion across trials (BL to US4), and analyzed with a mixed-factor ANOVA (age × genotype × sex × trial). To further account for the possibility that habituation may confound differences in %time freezing, the relative change in motion between baselines on all days was analyzed using a mixed-factor ANOVA (age × genotype × sex × day). Finally, the prolonged stress of fear conditioning has been shown to initiate weight loss because of the inhibition of consummatory behavior in rats (Pare, 1965). As an additional measure to account for habituation, the weight of each rat (in grams) was analyzed across days using a multifactor ANOVA (age × genotype × sex × day).

#### Extracellular fEPSPs

In all electrophysiology experiments, sex was underpowered and therefore excluded as a factor. In the I/O experiments, fEPSP peak amplitude across stimulation intensity was analyzed using a mixed-factor ANOVA (age × genotype × intensity) with age (three levels) and genotype (two levels) as between-subjects factors, and intensity as the within-subjects factor (17 levels: 20–100 μA in 5 μA steps). Main effects, interactions, and a second tier of analyses (genotype × intensity at each age) were followed up as stated above. For each fEPSP recorded in the I/O experiments, a coastline burst index (CBI) was generated to assess possible hyperexcitability (Korn et al., 1987; Stewart et al., 2017; Widman and McMahon, 2018). To leverage the full design of each ANOVA, missing data points for I/O experiments in *n* = 1 young adult WT rat and *n* = 1 young adult TgAD rat were computed by linear interpolation using the missing data tool in SPSS (note that effects and interpretations were not influenced by missing data). For LTP experiments, peak amplitude was transformed to the percentage of baseline, and the initial analysis used a mixed-factor ANOVA (age × genotype × time), with age and genotype as described above and time as the within-subjects factor (40 levels: minutes −1 to 30). Pairwise comparisons were used as stated above, and a follow-up genotype × time ANOVA was used within each age group, as stated above. In addition, to determine whether individual groups were successful at short-term potentiation (STP) or LTP, paired-samples *t* tests were used to compare peak amplitudes (as %BL) during the first 5 min post-HFS to BL (STP) and to compare peak amplitudes (as %BL) at 30 min post-HFS to BL (LTP) for each group separately.

#### Extracellular fEPSP control measures

To account for day-to-day variation during I/O experiments, CBIs during the prestimulus period from each recording (first 20 ms) were compared using a mixed-factor ANOVA (age × genotype × intensity). To account for potential baseline differences confounding the effects of age or genotype in LTP experiments, baselines were compared using a mixed-factor ANOVA (age × genotype × time).

#### Principal component analysis

For all rats with behavioral and electrophysiological data, a PCA was used to analyze associations between fear memory extinction and BA synaptic function. Standardized scores for percentage of time freezing during Mem, Ext, and Ret were loaded to represent CS memory, extinction, and extinction memory retrieval, respectively. Standardized scores of I/O peak amplitudes and peak amplitudes 30 min post-HFS were loaded to represent synaptic physiology. To avoid split loadings, the rotation used was Varimax with Kaiser normalization. An eigenvalue >1 was considered meaningful, and factor loadings >0.6 were considered significant. Each component score was extracted as regression loadings, and the unique loadings for each animal were used to plot its distribution on each component. To determine group differences, regression loadings were analyzed using a multivariate ANOVA (age × genotype) with each component as a dependent variable (DiStefano et al., 2009). Finally, using a combination of SPSS and R (ggplot2), regression loadings were used to visualize group clustering within the rotated space, and 95% confidence ellipses were generated using the ellipse stat function.

## Results

### Effects of age and AD on fear memory

#### Fear memory acquisition

**Figure 2.**
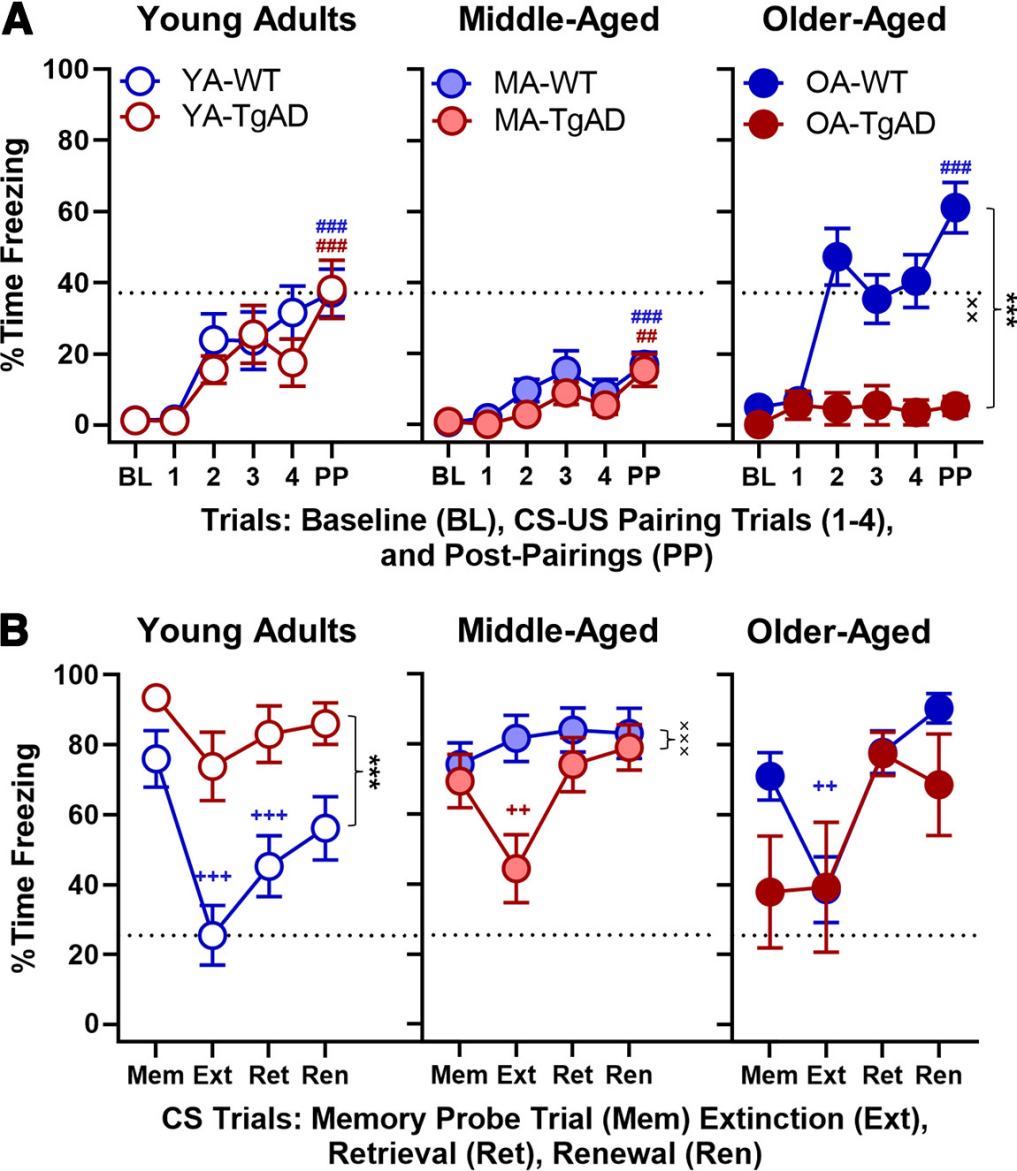
Acquisition, extinction, retrieval, and renewal. ***A***, Day 1 acquisition. There were no genotype differences within the young adult or middle-aged groups. Within the older-aged group, there was a significant main effect of genotype and a significant genotype × trial interaction such that WT showed greater %time freezing relative to TgAD. With the exception of older-aged TgAD rats, all groups acquired fear memory during day 1. The *x*-axis represents BL, CS–US paring trials (1–4), and PPs. ***B***, Days 2–4 extinction, retrieval, and renewal. Within the young adults, there was a main effect of genotype such that TgAD rats showed greater %time freezing relative to WT. While young adult WT rats showed successful fear extinction and retrieval, young adult TgAD rats did not. Within the middle-aged group, there was a significant genotype × trial interaction. Middle-aged TgAD rats showed intact acute fear extinction but not retrieval, whereas the WT middle-aged rats did not show either. There were no genotypic differences within the older-aged group. Older-aged WT rats showed intact acute fear extinction but not retrieval, whereas there was no extinction or retrieval in the older-aged TgAD rats. The x-axis represents the memory probe trial (Mem), extinction (Ext), retrieval (Ret), and renewal (Ren). Data are plotted as the mean with SEM. ***p < 0.001 for main effects of genotype; ××*p* < 0.01, ×××*p* < 0.001 for genotype × intensity interactions; ++*p* < 0.01, +++*p* < 0.001 for significant differences between Ext or retrieval and Mem within groups (blue for WT and red for TgAD); ##*p* < 0.01, ###*p* < 0.001 for significant differences between BL and PP during acquisition within groups (WT, blue; TgAD, red). Dotted black line represents mean of YA-WT PP in ***A*** and mean of YA-WT Ext in ***B***.

We first tested whether differences exist between WT and TgAD rats in their ability to acquire fear memory over the life span. On day 1, young adult, middle-aged, and older-aged WT and TgAD rats received CS–US pairings after a baseline period in context A. Two outliers (*n* = 1 young adult WT rat; *n* = 1 middle-aged TgAD rat) were identified and removed from this and all subsequent analyses. Aging affected %time freezing (*F*_(2,87)_ = 9.765, *p* < 0.001, η_p_^2^ = 0.183, 1-β = 0.980) such that middle-aged rats froze significantly less compared with young rats (−11.460, *p* < 0.001) and older rats (−9.063; *p* = 0.008); all TgAD rats froze less (−9.447) relative to WT rats (*F*_(1,87)_ = 12.979, *p* < 0.001, η_p_^2^ = 0.130, 1-β = 0.945); all males froze less (−6.005) relative to females (males: 10.549 ± 1.807; females: 16.553 ± 1.901; *F*_(1,87)_ = 5.243, *p* = 0.024, η_p_^2^ = 0.057, 1-β = 0.620); and all rats froze more in all trials after CS–US1 relative to baseline (+14.676–25.763; *F*_(5,435)_ = 22.638, *p* < 0.001, η_p_^2^ = 0.206, 1-β = 1.000). Furthermore, there were significant age × genotype (*F*_(2,87)_ = 5.093, *p* = 0.008, η_p_^2^ = 0.105, 1-β = 0.809), age × trial (*F*_(10,435)_ = 2.369, *p* = 0.013, η_p_^2^ = 0.052, 1-β = 0.918), and genotype × trial (*F*_(5,435)_ = 2.535, *p* = 0.033, η_p_^2^ = 0.028, 1-β = 0.756) interactions. All other interactions were nonsignificant (*F* values_(1–10,87–435)_ = 0.210–2.746, *p* values = 0.101–0.957; Fig. 2*A*).

To better define the nature of these interactions and to test whether the degree of acquisition was different between WT and TgAD rats at each age, we followed up with a genotype × trial ANOVA. Within the young adult age group, genotypes were not different in any trial (*F* values_(1–5,29–145)_ = 0.407–0.813, *p* values = 0.509–0.528). However, all young adults progressively froze more after baseline (trial: *F*_(5,145)_ = 16.352, *p* < 0.001, η_p_^2^ = 0.361, 1-β = 1.000; Fig. 2*A*). Similarly, within the middle-aged group, genotypes were not different at any trial (*F* values_(1–5,39–195)_ = 0.546–1.556, *p* values = 0.220–0.673), and all rats progressively froze more after baseline (trial: *F*_(5,195)_ = 10.727, *p* < 0.001, η_p_^2^ = 0.216, 1-β = 1.000; Fig. 2*A*). In contrast, older-aged TgAD rats froze less after baseline relative to their WT counterparts (genotype: *F*_(1,25)_ = 21.507, *p* < 0.001, η_p_^2^ = 0.462, 1-β = 0.994; genotype × trial: *F*_(5,125)_ = 3.910, *p* = 0.003, η_p_^2^ = 0.135, 1-β = 0.928; trial: *F*_(5,125)_ = 4.668, *p* < 0.001, η_p_^2^ = 0.157, 1-β = 0.966; Fig. 2*A*).

Finally, to confirm that all WT and TgAD rats acquired fear memory, we used paired-samples *t* tests to compare %time freezing during baseline to %time freezing during PPs. Figure 2*A* shows increased freezing from baseline to postpairings, confirming fear memory acquisition in young adult WT rats (*t*_(15)_ = 5.476, *p* < 0.001, *d* =* *1.369), young adult TgAD rats (*t*_(14)_ = 4.492, *p* < 0.001, *d* =* *1.160), middle-aged WT rats (*t*_(19)_ = 4.611, *p* < 0.001, *d* =* *1.031), middle-aged TgAD rats (*t*_(20)_ = 3.082, *p* = 0.006, *d* =* *0.672), older-aged WT rats (*t*_(20)_ = 8.168, *p* < 0.001, *d* =* *1.782), but not older-aged TgAD rats (*t*_(5)_ = 1.932, *p* = 0.111, *d* =* *0.789).

Collectively, these results suggest varying degrees of intact fear memory acquisition in all rats except older-aged TgAD rats. It should be noted that, in contrast to previous reports (Maren et al., 1994; Pryce et al., 1999), we found freezing time during acquisition was modestly greater in females, which is consistent with females being more sensitive to punishment (Miettunen et al., 2007; Orsini et al., 2016; Liley et al., 2019; Hernandez et al., 2020). Furthermore, female 3×TgAD and control mice have greater freezing relative to males (Stover et al., 2015). Notably, these sex differences do not explain the age and genotype effects on acquisition.

#### Fear memory extinction, retrieval, and renewal

We then tested whether fear memory of the CS was intact within-groups on day 2 by using paired-samples *t* tests to compare %time freezing between baseline (Table 2) and the CS memory probe (Mem) trial. These results revealed young adult WT rats (*t*_(15)_ = 7.779, *p* < 0.001, *d* = 1.945), young adult TgAD rats (*t*_(14)_ = 4.993, *p* < 0.001, *d* = 1.289), middle-aged WT rats (*t*_(19)_ = 5.808, *p* < 0.001, *d* = 1.299), middle-aged TgAD rats (*t*_(20)_ = 6.687, *p* < 0.001, *d* = 1.459), and older-aged WT rats (*t*_(20)_ = 7.669, *p* < 0.001, *d* = 1.673) increased freezing, suggesting robust expression of fear memory to the CS. The older-aged TgAD rats did not show an increase in %time freezing between baseline and the probe CS memory trial (*t*_(5)_ = 0.679, *p* = 0.527). However, to address whether the null effect was confounded by an increase in %time freezing during baseline, a comparison of %time freezing between the final CS trial during acquisition and memory probe trial (Mem) revealed a significant increase in fear memory expression (*t*_(5)_ = 2.044, *p* = 0.048, *d* = 0.834).

**Table 2:** Percentage of time freezing during baseline on day 2

Genotype	Age	Mean	SEM
WT	Young adult	37.228	6.709
	Middle-aged	39.195	5.291
	Older-aged	33.283	5.571
TgAD	Young adult	57.408	6.767
	Middle-aged	36.558	6.252
	Older-aged	27.928	7.710

After confirming there was fear memory in all groups, we tested whether differences exist between WT and TgAD rats over the life span in fear memory extinction, retrieval, and renewal. Rats received CS trials without the US pairing in a novel context B to induce extinction on day 2. Then, we tested fear extinction memory retrieval in WT and TgAD rats over the life span on day 3. Finally, all rats were given CS trials without US pairings in context A to test for fear memory renewal on day 4. While an analysis of %time freezing during extinction, extinction memory retrieval, and renewal using a mixed-factor ANOVA (age × genotype × sex × trial) did not reveal any main effects of age, genotype, or sex (*F* values_(1–2,87)_ = 0.000–1.549, *p* values = 0.218–0.996), the main effect of trial was significant (*F*_(3,261)_ = 21.423, *p* < 0.001, η_p_^2^ = 0.198, 1-β = 1.000). Furthermore, there were significant age × genotype (*F*_(2,87)_ = 9.529, *p* < 0.001, η_p_^2^ = 0.180, 1-β = 0.977), age × trial (*F*_(6,261)_ = 5.724, *p* < 0.001, η_p_^2^ = 0.116, 1-β = 0.997), and age × genotype × trial (*F*_(6,261)_ = 4.219, *p* < 0.001, η_p_^2^ = 0.088, 1-β = 0.978) interactions (Fig. 2*B*). All other interactions were nonsignificant (*F* values_(1–6,87–261)_ = 0.382–2.128, *p* values = 0.097–0.766).

To better understand the interaction, we tested whether freezing during extinction, retrieval, and renewal differed between WT and TgAD groups at each age. Within the young adult age group, TgAD rats froze more overall relative to WT (genotype: *F*_(1,29)_ = 16.523, *p* < 0.001, η_p_^2^ = 0.363, 1-β = 0.975; genotype × trial: *F*_(3,87)_ = 2.128, *p* = 0.103; trial: *F*_(3,87)_ = 10.723, *p* < 0.001, η_p_^2^ = 0.270, 1-β = 0.999; Fig. 2*B*). Within the middle-aged group, genotype interacted with trial (*F*_(3,117)_ = 6.106, *p* < 0.001, η_p_^2^ = 0.135, 1-β = 0.956) such that WT rats froze more (+37.229, *p* = 0.003) only during Ext relative to TgAD (trial: *F*_(3,117)_ = 6.638, *p* < 0.001, η_p_^2^ = 0.145, 1-β = 0.970; genotype: *F*_(1,39)_ = 2.534, *p* = 0.120; Fig. 2*B*). In the older-aged group, genotypes were not different at any trial (*F* values_(1–3,25–75)_ = 1.632–1.915, *p* values = 0.179–0.189) and all rats froze more during renewal testing (*F*_(3,75)_ = 9.013, *p* < 0.001, η_p_^2^ = 0.265, 1-β = 0.994).

We then tested whether WT and TgAD rats expressed fear memory extinction, retrieval, and renewal across the life span with paired-samples *t* tests. Young adult WT rats decreased freezing during extinction (*t*_(15)_ = −5.294, *p* < 0.001, *d* =* *1.324; Fig. 2*B*) and retrieval relative to the memory probe trial (*t*_(15)_ = −4.529, *p* < 0.001, *d* =* *1.132), but not during renewal relative to retrieval (*t*_(15)_ = 1.427, *p* = 0.174), suggesting there was intact extinction and extinction memory retrieval, whereas the lack of renewal may indicate some resilience to expressing fear as a result of maintained fear extinction memory. Young adult TgAD rats only tended to slightly decrease freezing during extinction (*t*_(14)_ = −2.095, *p* = 0.055, *d* =* *0.541; Fig. 2*B*) with no other differences (*t* values_(14)_ = 0.488–1.110, *p* values = 0.286–0.633), suggesting an early life impairment in fear extinction. Middle-aged WT rats showed no differences between trials (*t* values_(19)_ = 0.170–1.590, *p* values = 0.128–0.867). Surprisingly, middle-aged TgAD rats did decrease freezing during extinction (*t*_(20)_ = −2.881, *p* = 0.009, *d* =* *0.629; Fig. 2*B*), but not during retrieval, and showed no other differences (*t* values_(20)_ = 0.873–1.351, *p* values = 0.192–0.393), suggesting a compensation in the ability to extinguish fear memory but not the ability to maintain that memory long term. Older-aged WT rats also decreased freezing during extinction (*t*_(20)_ = −3.077, *p* = 0.006, *d* =* *0.671; Fig. 2*B*), but not retrieval, and showed no other differences (*t* values_(20)_ = 1.324–1.741, *p* values = 0.097–0.200), suggesting a late-life reemergence in the ability to extinguish fear memory as a function of nonpathologic aging without maintenance of that extinction memory. Finally, older-aged TgAD rats showed no differences between trials (*t* values_(5)_ = 0.066–2.420, *p* values = 0.060–0.950).

These results show successful fear memory extinction and retrieval in the absence of fear renewal in the young adult WT rats, whereas the young adult TgAD rats were extinction impaired. Moreover, while middle-aged WT rats were extinction impaired, middle-aged TgAD rats surprisingly showed acute fear memory extinction, potentially resulting from an age-dependent compensatory mechanism that is not sustained into old age. However, middle-aged TgAD rats were impaired in the ability to retrieve fear extinction memory after a 24 h delay. Similarly, while the older-aged WT rats showed acute fear memory extinction, they were impaired at retrieval. Fear memory expression in older-aged TgAD rats did emerge during the memory probe trial, but instead of extinguishing fear memory, they showed increased fear memory expression during retrieval and renewal testing. The absence of fear renewal may be because of a ceiling effect in fear expression during retrieval. In the young adult WT rat, however, no significant renewal to the CS may be explained by either resilience against fear renewal or by slightly elevated fear expression during retrieval. As there was a numerical increase in fear memory expression from retrieval to renewal, it is more likely that the null effect occurs because of original fear memory savings during retrieval, and, given more extinction training, it is possible that the difference in fear memory expression between retrieval and renewal would become significant in young WT rats.

#### Fear memory to context

As fear memory to context and CS are dissociable, in a separate analysis we tested whether freezing to contextual cues alone differed between groups. While statistical analyses did not reveal any main effects of age, genotype, or sex (*F* values_(1–2,87)_ = 0.090–1.372, *p* values = 0.245–0.860), the main effect of trial was significant (*F*_(2,174)_ = 68.957, *p* < 0.001, η_p_^2^ = 0.442, 1-β = 1.000). Furthermore, there were age × genotype (*F*_(2,87)_ = 7.473, *p* = 0.001, η_p_^2^ = 0.147, 1-β = 0.935), age × trial (*F*_(4,174)_ = 6.854, *p* < 0.001, η_p_^2^ = 0.136, 1-β = 0.993), genotype × trial (*F*_(2,174)_ = 5.437, *p* = 0.005, η_p_^2^ = 0.059, 1-β = 0.842), and genotype × sex × trial (*F*_(2,174)_ = 3.890, *p* = 0.022, η_p_^2^ = 0.043, 1-β = 0.697) interactions. No other interactions were significant (*F* values_(1–4,87–174)_ = 0.016–1.854, *p* values = 0.132–0.984).

**Figure 3.**
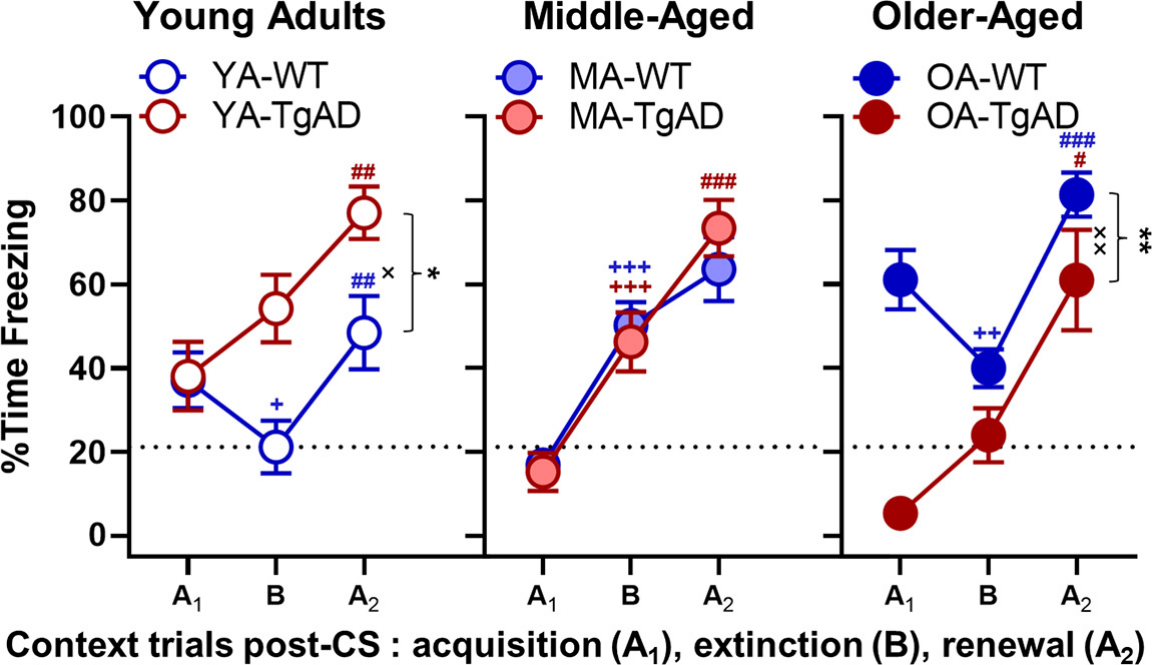
Context. Percentage of time freezing to different contextual conditions (context A postpairings during acquisition → context B → context A post-CS during renewal) within each age group. Within the young adults, there was a significant main effect of genotype and a significant genotype × trial interaction such that TgAD rats progressively increased %time freezing relative to WT rats. Within the young adult WT rats, there was decreased %time freezing from context A during acquisition to context *B*, and then increased freezing from context B to context A during renewal. Within the young adult TgAD rats, there was a significant increase in %time freezing from context B to context A during renewal. There were no genotypic differences in the middle-aged group. Within both WT and TgAD middle-aged rats, there was a progressive increase in %time freezing across context testing; however, the increase between context B and context A during renewal in middle-aged WT rats was not significant. Within the older-aged group, there was a significant main effect of genotype and a significant genotype × trial interaction such that freezing was greater in WT rats relative to TgAD rats. Older-aged WT rats showed a decrease in %time freezing between context A during acquisition and context *B*, and an increase between context B and context A during renewal. In contrast, older-aged TgAD rats showed a significant increase in %time freezing between context B and context A during renewal. Data are plotted as the mean with SEM. *p < 0.05, **p < 0.01 for main effects of genotype; ×*p* < 0.05, ××*p* < 0.01 for genotype × intensity interactions; +*p* < 0.05, ++*p* < 0.01, +++*p* < 0.001 for significant differences between context A during acquisition and context B within groups (WT, blue; TgAD, red); ##*p* < 0.01, ###*p* < 0.001 for significant differences between context B and context A during renewal within groups. Dotted black line represents the mean of YA-WT PP for context B.

We then tested whether freezing to context differed between genotype groups at each age in a follow-up analysis. Within the young adults WT froze less during context B and A_2_ testing relative to their TgAD counterparts (genotype: *F*_(1,29)_ = 6.306, *p* = 0.018, η_p_^2^ = 0.179, 1-β = 0.680; trial: *F*_(2,58)_ = 13.632, *p* < 0.001, η_p_^2^ = 0.320, 1-β = 0.997; genotype × trial: *F*_(2,58)_ = 4.898, *p* = 0.011, η_p_^2^ = 0.144, 1-β = 0.785; Fig. 3), consistent with the interpretation that TgAD rats generalized contexts. Within the middle-aged group, there were no genotypic differences at any trial (*F* values_(1–2,39–78)_ = 0.048–1.101, *p* values = 0.338–0.828), but all rats progressively froze more across context testing (*F*_(2,78)_ = 57.083, *p* < 0.001, η_p_^2^ = 0.144, 1-β = 0.785). Within the older-aged group, WT rats froze more during all of context testing (genotype: *F*_(1,25)_ = 12.930, *p* = 0.001, η_p_^2^ = 0.341, 1-β = 0.932; trial: *F*_(2,50)_ = 21.705, *p* < 0.001, η_p_^2^ = 0.465, 1-β = 1.000; genotype × trial: *F*_(2,50)_ = 5.194, *p* = 0.009, η_p_^2^ = 0.172, 1-β = 0.806; Fig. 3).

Finally, we tested whether WT and TgAD rats at all ages could discriminate between safe and unsafe contexts with paired-samples *t* tests. Young adult WT rats froze less to context B relative to context A_1_ (*t*_(15)_ = −2.135, *p* = 0.0496, *d* = −0.534; Fig. 3), indicating less fear for context B, and when they were placed back into context A_2_ during renewal, there was an expected freezing increase relative to context B (*t*_(15)_ = 3.669, *p* = 0.002, *d* =* *0.917; Fig. 3), demonstrating fear renewal to the unsafe context. In contrast, young adult TgAD rats showed a nonsignificant freezing increase from context A_1_ to B (*t*_(14)_ = 1.789, *p* = 0.095) and a significant increase from context B to A_2_ (*t*_(15)_ = 3.194, *p* = 0.007, *d* =* *0.825; Fig. 3) consistent with the inability to discriminate safe and unsafe contextual cues. Similarly, both middle-aged WT and TgAD rats showed progressive increases in freezing from context A_1_ to context B (WT: *t*_(19)_ = 5.925, *p* < 0.001, *d* =* *1.325; TgAD: *t*_(20)_ = 4.090, *p* < 0.001, *d* =* *0.893; Fig. 3) and from context B to context A_2_ (WT: *t*_(19)_ = 2.057, *p* = 0.054, *d* =* *0.460; TgAD: *t*_(20)_ = 4.522, *p* < 0.001, *d* =* *0.987; Fig. 3). Older-aged WT rats decreased freezing between context A_1_ and context B (*t*_(20)_ = −2.960, *p* = 0.008, *d* = −0.646; Fig. 3) and then increased freezing between context B and context A_2_ (*t*_(20)_ = 7.741, *p* < 0.001, *d* =* *1.630; Fig. 3), suggesting the reemergence of some ability to discriminate contextual cues. However, older-aged WT rats expressed greater fear during the safe context relative to their young counterparts (*t*_(35)_ = 2.493, *p* = 0.018, *d* =* *0.827), indicating aging is associated with maladaptive responses in emotional regulation. In contrast, older-aged TgAD rats tended to increase freezing between context A_1_ and context B (*t*_(5)_ = 2.374, *p* = 0.064, *d* =* *0.969) and significantly freezing between context B and context A_2_ (*t*_(5)_ = 3.660, *p* = 0.015, *d* =* *1.494; Fig. 3). Together, these data suggest that context discernment and renewed fear memory is intact in young adult WT rats, whereas all other groups showed greater freezing and impairments such that unsafe and safe context were grossly generalized.

#### Behavioral control measures

To determine whether shock perception (reactivity) partially explained any effects of age and genotype on freezing across testing phases, we analyzed the change in motion across trials (as defined in Materials and Methods). There was an expected increase in motion between baseline and US1 that was maintained across successive trials (*F*_(3,261)_ = 132.407, *p* < 0.001, η_p_^2^ = 0.603, 1-β = 1.000), and females responded slightly more (0.299 ± 0.145 in pixel change) than males (sex: *F*_(1,87)_ = 4.228, *p* = 0.043, η_p_^2^ = 0.046, 1-β = 0.529; sex × trial: *F*_(3,261)_ = 7.055, *p* = 0.001, η_p_^2^ = 0.075, 1-β = 0.929). However, as noted above, sex as a biological factor did not explain age and genotype effects on extinction, extinction memory retrieval, and renewal. Importantly, there were no effects of age, genotype, or any other interactions on shock perception (*F* values_(1–6,87–261)_ = 0.056–2.036, *p* values = 0.157–0.866). As such, differences in shock perception do not explain the effects of age and genotype on fear extinction, retrieval, and renewal (Table 3).

**Table 3:** Behavioral control measures

Measure	Age	Independent variable	Genotype			
			WT		TgAD	
			Mean	SE	Mean	SE
Change in shock reactivity	Young adult	CSUS1 - BL	3.756	0.544	3.894	0.563
		CSUS2 - CSUS1	−0.138	0.065	−0.041	0.067
		CSUS3 - CSUS2	1.127	0.432	−0.037	0.447
		CSUS4 - CSUS3	−0.042	0.072	0.135	0.075
	Middle-aged	CSUS1 - BL	4.582	0.496	3.305	0.475
		CSUS2 - CSUS1	−0.098	0.059	−0.099	0.057
		CSUS3 - CSUS2	0.132	0.394	0.031	0.377
		CSUS4 - CSUS3	0.022	0.066	0.073	0.063
	Older-aged	CSUS1 - BL	4.101	0.557	3.759	0.942
		CSUS2 - CSUS1	0.047	0.067	−0.152	0.113
		CSUS3 - CSUS2	0.022	0.442	0.123	0.748
		CSUS4 - CSUS3	−0.094	0.074	−0.064	0.125
Change in baseline motion	Young adult	Day2 - Day1	−0.572	0.060	−0.825	0.062
		Day3 - Day2	0.751	4.850	15.013	5.020
		Day4 - Day3	−0.007	0.718	3.464	0.743
	Middle-aged	Day2 - Day1	−0.838	0.054	−0.669	0.052
		Day3 - Day2	−0.170	4.428	−0.484	4.238
		Day4 - Day3	0.430	0.655	0.436	0.627
	Older-aged	Day2 - Day1	−0.685	0.061	−0.685	0.103
		Day3 - Day2	−0.165	4.970	0.047	8.401
		Day4 - Day3	0.125	0.735	−0.123	1.243
Body weights (g)	Young adult	day 1	356.625	8.570	368.902	8.871
		day 2	354.000	8.543	367.598	8.843
		day 3	354.188	8.621	365.000	8.923
		day 4	352.875	8.657	364.429	8.961
	Middle-aged	day 1	422.792	7.824	432.573	7.489
		day 2	420.833	7.799	431.059	7.466
		day 3	419.813	7.869	431.455	7.533
		day 4	418.396	7.903	431.295	7.565
	Older-aged	day 1	360.369	8.782	412.750	14.844
		day 2	359.213	8.754	409.125	14.797
		day 3	356.819	8.833	408.000	14.931
		day 4	356.013	8.871	404.500	14.995

Mean and SE of each measure for young, middle-aged, and older-aged WT and TgAD rats.

We then wanted to determine whether habituation partially explained any effects of age and genotype on freezing across testing phases. Therefore, we analyzed the relative change in motion in baselines across days and confirmed no main effects or interactions (*F* values_(1–4,87–174)_ = 0.784–1.670, *p* values = 0.199–0.475). Moreover, an analysis of body weight across days revealed that all rats consistently lost weight from day 1 to day 4 (*F*_(3,261)_ = 25.645, *p* < 0.001, η_p_^2^ = 0.228, 1-β = 1.000), suggesting a stress-driven inhibition of consummatory behavior during contextual fear conditioning (Pare, 1965; Table 3). Additionally, TgAD rats weighed more than WT rats (*F*_(1,87)_ = 9.578, *p* = 0.003, η_p_^2^ = 0.099, 1-β = 0.864; TgAD, 402.22 ± 6.282 g; WT, 377.66 ± 4.851 g). An age effect (*F*_(2,87)_ = 33.017, *p* < 0.001, η_p_^2^ = 0.431, 1-β = 1.000) revealed that young adults weighed less relative to other age groups (young adults, 360.452 ± 6.167 g; middle-aged, 426.027 ± 5.415 g; older-aged, 383.348 ± 8.624 g; *p* values = 0.034 to <0.001). Finally, females, as expected, weighed less than males (sex: *F*_(1,87)_ = 456.256, *p* < 0.001, η_p_^2^ = 0.840, 1-β = 1.000; males, 474.706 ± 5.468 g; females, 305.179 ± 5.757 g; interactions with sex: *F* values_(1–3,87–261)_ = 3.143–15.131, *p* values = 0.031 to <0.001, η^2^ values = 0.035–0.184, 1-β values = 0.687–0.980). No other interactions were significant (*F* values_(2–6,87–261)_ = 0.954–2.087, *p* values = 0.072–0.450). The null effects on change in motion across days coupled with a day-dependent decrease in weight as a marker of stress suggest that the effects of age and genotype on %time freezing during fear extinction, retrieval, and renewal were not explained by habituation.

### Effects of age and AD on basolateral amygdala synaptic physiology

#### Basal synaptic strength

**Figure 4.**
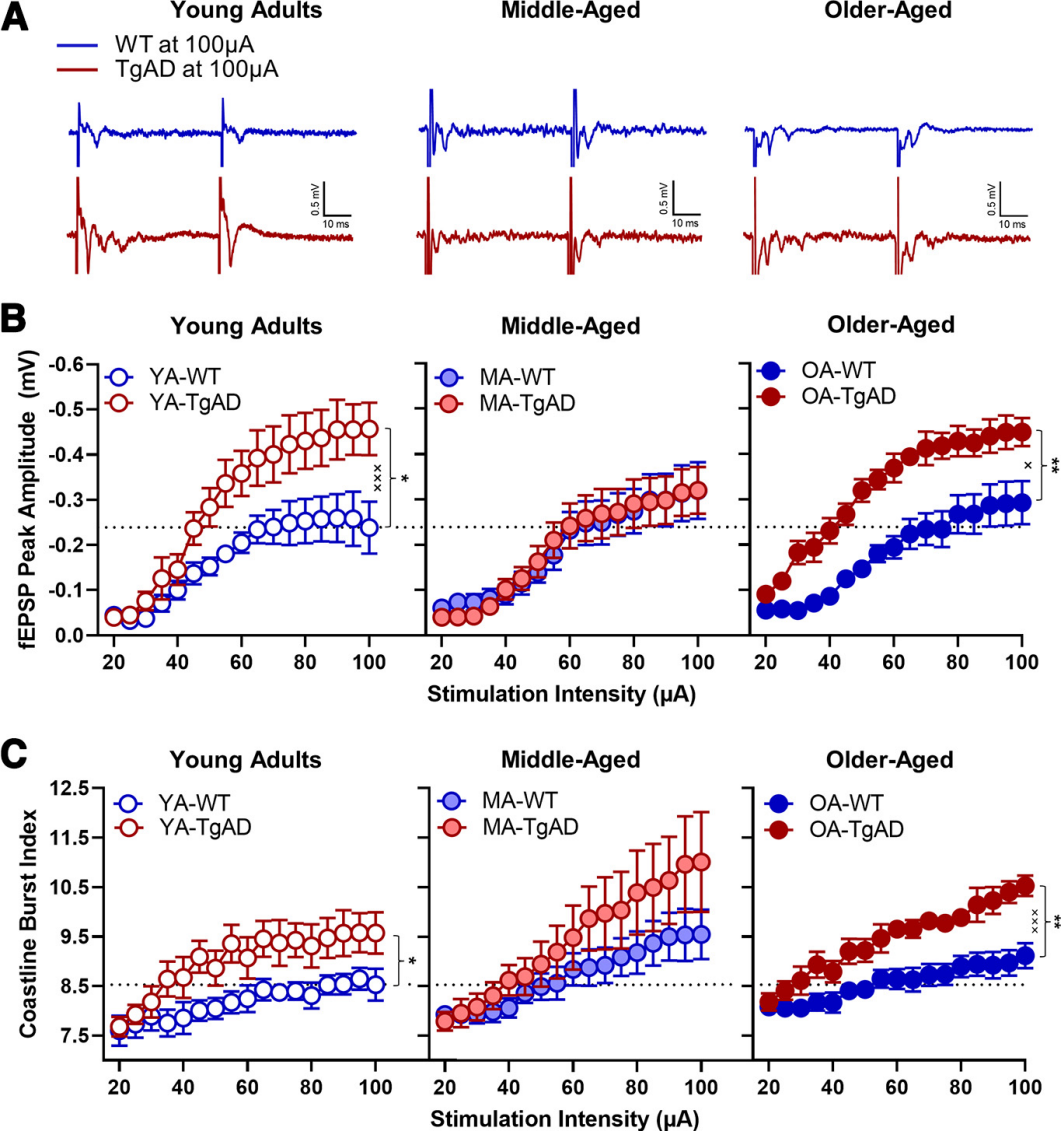
fEPSP peak amplitude responses to increasing stimulation intensity. ***A***, Representative traces for each group at maximum simulation intensity (100 μA). ***B***, fEPSP peak amplitudes within age groups. Within the young adults, there was a significant main effect of genotype and a significant genotype × intensity interaction such that TgAD rats had greater peak amplitudes at higher stimulation intensities relative to WT rats. There were no differences between WT and TgAD rats within the middle-aged group. Within the older-aged group, there was a significant main effect of genotype and a significant genotype × intensity interaction such that TgAD rats had greater peak amplitudes at higher stimulation intensities relative to WT rats. There was also a significant age × genotype interaction. ***C***, The coastline burst index for each age group. Within the young adults, there was a significant main effect of genotype such that TgAD rats had greater BA activity. There were no genotypic differences among the middle-aged rats. Within the older-aged group, there was a significant main effect of genotype and a significant genotype × intensity interaction such that older-aged TgAD rats had greater activity at higher stimulation intensities relative to WT rats. There was also a trending age × intensity interaction regardless of genotype. Data are plotted as the mean with SEM. *p < 0.05, **p < 0.01 for main effects of genotype; ×*p* < 0.05, ×××*p* < 0.001 for genotype × intensity interactions. Dotted black line represents mean of YA-WT at 100 μA.

Lesion and *in vivo* electrophysiology studies show that the BLA supports the associative learning between CS and US necessary for fear memory acquisition (LeDoux et al., 1990; Romanski et al., 1993). More specifically, localized lesions or protein synthesis blockage in the lateral amygdaloid nucleus (LA) impairs delay and contextual fear memory consolidation, whereas blocking protein synthesis in the BA prevents contextual fear memory consolidation (Kochli et al., 2015). Importantly, BA lesions do not block fear memory acquisition, whereas LA lesions do (Amorapanth et al., 2000). As acquisition was not grossly impaired by age or genotype, but fear extinction memory was, the behavioral differences observed could be explained by changes in synaptic function at excitatory synapses in the BA nucleus. Thus, we asked whether differences exist in the strength of basal excitatory synaptic transmission in the BA of WT and TgAD rats by recording extracellular fEPSPs in acute slices and performing stimulus–response curves (I/O curves; Fig. 4*A*). We measured peak fEPSP amplitude while increasing the stimulus intensity at 5 μA increments. While peak fEPSP amplitudes did not differ by age (*F*_(2,46)_ = 1.787, *p* = 0.179), peak fEPSP amplitudes in all TgAD rats were significantly greater relative to all WT rats (+0.091 mV, *F*_(1,46)_ = 11.987, *p* = 0.001, η_p_^2^ = 0.207, 1-β = 0.924; Fig. 4*B*), and all peak amplitudes increased at higher stimulation intensities, as expected (*F*_(16,736)_ = 98.820, *p* < 0.001, η_p_^2^ = 0.682, 1-β = 1.000). Although there were significant age × genotype (*F*_(2,46)_ = 1.787, *p* = 0.049, η_p_^2^ = 0.123, 1-β = 0.585) and genotype × intensity (*F*_(16,736)_ = 3.304, *p* = 0.0496, η_p_^2^ = 0.067, 1-β = 0.564) interactions, no other interactions were significant (*F* values_(2–32,46–736)_ = 0.711–0.856, *p* values = 0.479–0.565).

We followed up this analysis by testing whether the I/O curves differed by genotype at each age. Within the young adults, TgAD rats showed larger peak amplitudes at higher intensities relative to WT rats (genotype: *F*_(1,15)_ = 8.017, *p* = 0.013, η_p_^2^ = 0.348, 1-β = 0.754; intensity: *F*_(16,240)_ = 34.426, *p* < 0.001, η_p_^2^ = 0.697, 1-β = 1.000; genotype × intensity: *F*_(16,240)_ = 2.952, *p* < 0.001, η_p_^2^ = 0.164, 1-β = 0.998; Fig. 4*B*). Within the middle-aged group, there were no genotypic differences (*F* values_(1–16,19–304)_ = 0.002–0.268, *p* values = 0.708–0.962), but peak amplitudes increased at higher stimulation intensities in all rats (*F*_(16,304)_ = 32.046, *p* < 0.001, η_p_^2^ = 0.628, 1-β = 1.000; Fig. 4*B*). Within the older-aged group, TgAD rats showed larger peak amplitudes at higher intensities relative to WT (genotype: *F*_(1,12)_ = 15.399, *p* = 0.002, η_p_^2^ = 0.562, 1-β = 0.949; intensity: *F*_(16,192)_ = 52.101, *p* < 0.001, η_p_^2^ = 0.813, 1-β = 1.000; genotype × intensity: *F*_(16,192)_ = 52.101, *p* = 0.021, η_p_^2^ = 0.138, 1-β = 0.952; Fig. 4*B*).

#### Paired-pulse ratio

To determine whether the differences in the strength of basal transmission could be a consequence of differences in presynaptic release probability, we analyzed the paired-pulse ratio (PPR), an indirect measure of release probability (Dobrunz and Stevens, 1997). Analyzing the PPR at increasing stimulus intensities revealed that, regardless of age and genotype, facilitation increased from 0.036 to 0.121 between 20 and 40 μA, then decreased from 0.099 to 0.000 between 45 and 60 μA before it reversed to depression from −0.035 to −0.114 between 65 to 100 μA (*F*_(16,763)_ = 14.550, *p* < 0.001, η_p_^2^ = 0.240, 1-β = 1.000; Table 4). No other effects were significant (*F* values_(1–32,46–736)_ = 0.298–1.750, *p* values = 0.109–0.588). These findings indicate that presynaptic release probability does not explain the differences observed in the I/O curves and that the heightened transmission in the TgAD rats is unlikely to be caused by enhanced presynaptic glutamate release.

#### Hyperexcitability

To determine whether the heightened strength in basal transmission in young and aged TgAD rats is a consequence of hyperexcitability since there is no increase in presynaptic release probability, we analyzed the fEPSP traces using the CBI. The value of the index (in arbitrary units) is associated with the intensity of the underlying activity in the fEPSP, as previously reported (Korn et al., 1987; Stewart et al., 2017; Widman and McMahon, 2018). While CBIs did not differ by age (*F*_(2,46)_ = 0.913, *p* = 0.409, η_p_^2^ = 0.038), all TgAD rats had larger CBIs relative to all WT (+0.808; *F*_(1,46)_ = 6.491, *p* = 0.014, η_p_^2^ = 0.124, 1-β = 0.703), and CBIs increased in all rats at larger stimulation intensities (*F*_(16,736)_ = 41.445, *p* < 0.001, η_p_^2^ = 0.474, 1-β = 1.000), as expected when more axons are recruited at stronger stimulus intensities. Furthermore, there were significant genotype × intensity (*F*_(16,736)_ = 4.175, *p* = 0.020, η_p_^2^ = 0.083, 1-β = 0.712) and trending age × intensity (*F*_(32,736)_ = 2.455, *p* = 0.053, η_p_^2^ = 0.096, 1-β = 0.670) interactions with no other effects (*F* values_(2–32,46–736)_ = 0.040–0.405, *p* = 0.799–0.960).

To better understand the reported interactions above, we tested whether CBIs differed by genotype at each age. Indeed, within the young adult age, TgAD rats had larger CBIs at increasing intensities (genotype: *F*_(1,15)_ = 5.202, *p* = 0.038, η_p_^2^ = 0.257, 1-β = 0.569; intensity: *F*_(16,240)_ = 13.153, *p* < 0.001, η_p_^2^ = 0.467, 1-β = 1.000; genotype × intensity: *F*_(16,240)_ = 1.681, *p* = 0.051, η_p_^2^ = 0.101, 1-β = 0.919; Fig. 4*C*). Within the middle-aged group, genotypes did not differ (*F* values_(1–16,19–304)_ = 1.059–1.895, *p* values = 0.177–0.316), but CBIs increased with increasing intensity in all rats as expected with greater recruitment of axons (*F*_(16,304)_ = 19.393, *p* < 0.001, η_p_^2^ = 0.505, 1-β = 0.998; Fig. 4*C*). Within the older-aged group, TgAD rats had larger CBIs with increasing intensity relative to WT (genotype: *F*_(1,12)_ = 9.895, *p* = 0.008, η_p_^2^ = 0.452, 1-β = 0.823; intensity: *F*_(16,192)_ = 49.361, *p* < 0.001, η_p_^2^ = 0.804, 1-β = 0.998; genotype × intensity: *F*_(16,192)_ = 5.794, *p* < 0.001, η_p_^2^ = 0.326, 1-β = 1.000; Fig. 4*C*).

To confirm that genotypic differences in baseline synaptic strength and hyperexcitability were not explained by differences in the prestimulus period, we analyzed CBIs during the prestimulus period. Although there were no effects of intensity, genotype, or interactions (*F* values_(1–32,46–736)_ = 0.039–0.946, *p* values = 0.489–0.951), there was a small-magnitude effect of age (*F*_(2,46)_ = 5.153, *p* = 0.010, η_p_^2^ = 0.183, 1-β = 0.801) such that middle-aged rats showed smaller prestimulus CBIs relative to young adult rats (−0.189, *p* = 0.017) and older-aged rats (−0.238, *p* = 0.006; data not shown). However, a small-magnitude decrease in the middle-aged CBI prestimulus period does not explain the observed increase in CBIs driven by age and genotype.

Together, these data suggest that the BA of TgAD rats is overall hyperexcitable. The null effects of genotype on BA activity in middle-aged rats are consistent with the ephemeral compensation in acute fear memory extinction observed in the middle-aged TgAD rats. These data suggest that the BA of TgAD rats is hyperexcitable during young adulthood and undergoes a compensatory mechanism during middle-age, but reverts to a hyperexcitable state in older-aged rats.

#### Long-term potentiation

**Figure 5.**
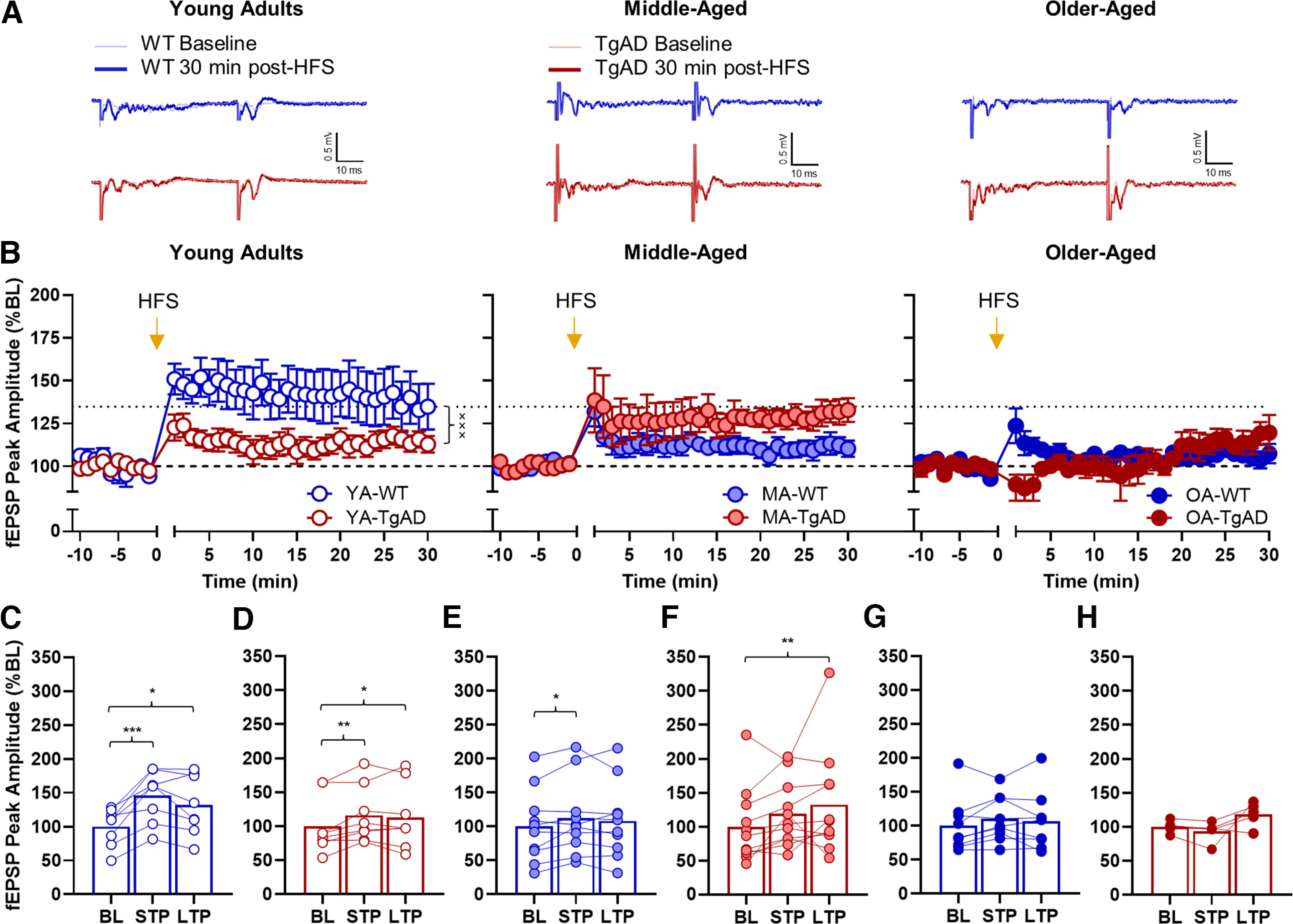
Effects of high-frequency stimulation on fEPSP peak amplitudes. ***A***, Representative traces for each group. ***B***, The fEPSP peak amplitude differences between baseline and post-HFS within each age group. There is a significant genotype × time interaction within the young adult group, whereas there are no genotypic differences within the other age groups. There was an overall main effect of age such that young adults had larger magnitude post-HFS peak amplitudes relative to middle-aged and older-aged groups. ***C***, STP and LTP relative to BL within the young adult WT rats. There was significant STP and LTP relative to BL. ***D***, STP and LTP relative to BL within the young adult TgAD rats. There was significant STP and LTP relative to BL. ***E***, STP and LTP relative to BL within the middle-aged WT rats. There was significant STP but not LTP relative to BL. ***F***, STP and LTP relative to BL within the middle-aged TgAD rats. While there was not significant STP, there was significant LTP relative to BL. ***G***, STP and LTP relative to BL within the older-aged WT rats. There was no STP or LTP relative to BL. ***H***, STP and LTP relative to BL within the older-aged TgAD rats. There was no STP or LTP relative to BL. Data are plotted as the mean and SEM and fEPSP peak amplitudes as percentage of baseline. *p < 0.05, **p < 0.01, ***p < 0.001 for paired-samples *t* tests; ×××*p* < 0.001 for genotype × time interactions. Dotted black line represents mean of YA-WT at 30 min post-HFS. Dashed black line represents baseline.

Given the changes in basal synaptic transmission, we next asked whether synaptic plasticity was inhibited in TgAD and WT rats across the life span. In Figure 5*A*, representative traces show peak amplitudes during baseline and post-HFS for each group. As reported previously (Zeng et al., 2012; Zhan et al., 2018), the maximum peak amplitude decreases with age (*F*_(2,45)_ = 4.036, *p* = 0.024, η_p_^2^ = 0.152, 1-β = 0.691) such that older-aged rats showed smaller peak amplitudes across all post-HFS time points relative to young adult (−16.394, *p* = 0.007) and middle-aged (−11.215, *p* = 0.049) rats (Fig. 5*B*). While there was no main effect of genotype (*F*_(1,45)_ = 0.571, *p* = 0.454), there was an expected effect of time (*F*_(39,1755)_ = 12.454, *p* < 0.001, η_p_^2^ = 0.217, 1-β = 1.000) indicating expected increases in peak amplitudes post-tetanus across all groups. Importantly, age interacted with all factors, whereas genotype did not interact with time (age × genotype: *F*_(2,45)_ = 4.766, *p* = 0.013, η_p_^2^ = 0.175, 1-β = 0.766; age × time: *F*_(78,1755)_ = 2.101, *p* = 0.029, η_p_^2^ = 0.085, 1-β = 0.874; age × genotype × time: *F*_(78,1755)_ = 2.079, *p* = 0.031, η_p_^2^ = 0.085, 1-β = 0.870; genotype × time: *F*_(39,1755)_ = 1.396, *p* = 0.231).

We then asked whether there were general magnitude differences between genotype groups at each age. Within the young adults, peak amplitudes at all time points after HFS were larger in WT rats relative to TgAD rats (genotype: *F*_(1,14)_ = 3.615, *p* = 0.078; time: *F*_(39,546)_ = 9.161, *p* < 0.001, η_p_^2^ = 0.396, 1-β = 1.000; genotype × time: *F*_(39,546)_ = 2.399, *p* < 0.001, η_p_^2^ = 0.146, 1-β = 1.000; Fig. 5*B*). Within the middle-aged group, though, there were numerical differences: the magnitude of peak amplitudes were not statistically different between WT and TgAD rats (*F* values_(1–39,19–741)_ = 1.042–3.316, *p* values = 0.084–0.402). However, peak amplitudes were greater at all timepoints after HFS relative to baseline in all middle-aged rats (time: *F*_(39,741)_ = 6.088, *p* < 0.001, η_p_^2^ = 0.243, 1-β = 1.000). Within the older-aged group, TgAD rats showed decreased peak amplitudes only within the first 3 min post-HFS relative to WT rats (genotype: *F*_(1,12)_ = 0.406, *p* = 0.536; time: *F*_(39,468)_ = 2.181, *p* < 0.001, η_p_^2^ = 0.154, 1-β = 1.000; genotype × time: *F*_(39,468)_ = 2.668, *p* < 0.001, η_p_^2^ = 0.182, 1-β = 1.000; Fig. 5*B*). Finally, these effects were not explained by baseline differences in peak amplitudes (age × genotype × time ANOVA: *F* values_(1–39,19–741)_ = 3.363–1.410, *p* values = 0.197–0.830).

In addition to testing group differences, we wanted to confirm the degree to which WT and TgAD rats across the life span were capable of STP and LTP by using paired-samples *t* tests. Young adult WT rats had larger peak amplitudes within the first 5 min (*t*_(7)_ = 5.525, *p* < 0.001, *d* =* *1.953) and also at 30 min (*t*_(7)_ = 2.414, *p* = 0.047, *d* =* *0.853) post-HFS (Fig. 5*C*). Young adult TgAD rats had larger peak amplitudes within the first 5 min (*t*_(7)_ = 3.761, *p* = 0.007, *d* =* *1.330) and also at 30 min (*t*_(7)_ = 2.929, *p* = 0.022, *d* =* *1.036) post-HFS (Fig. 5*D*). Middle-aged WT rats showed larger peak amplitudes within the first 5 min (*t*_(9)_ = 2.926, *p* = 0.017, *d* =* *0.925) but not at 30 min (*t*_(9)_ = 1.415, *p* = 0.191) post-HFS (Fig. 5*E*). Middle-aged TgAD rats did not show a difference in peak amplitudes within the first 5 min (*t*_(10)_ = 2.117, *p* = 0.060); however, peak amplitudes were larger at 30 min (*t*_(10)_ = 4.023, *p* = 0.002) post-HFS (Fig. 5*F*). In contrast to young adult and middle-aged rats, there were no differences between baseline and post-HFS peak amplitudes in older-aged WT rats (*t* values_(10)_ = 1.300–1.978, *p* values = 0.083–0.230; Fig. 5*G*) and TgAD (*t* values_(4)_ = −2.041–1.853, *p* values = 0.111–0.138; see Fig. 5*H*). Together, these data suggest that the attenuated LTP in young adult TgAD rats undergoes compensation during middle-age and is not sustain into older-age, whereas in WT rats aging significantly impairs BA LTP.

### Associations between behavior and synaptic physiology

The results above demonstrate extinction and extinction memory may rely on intact BA synaptic plasticity. As such, it is possible that measures of BA synaptic physiology can predict fear memory expression during phases of extinction testing. Therefore, a PCA was used to determine whether the individual variability in measures of synaptic physiology predicted the individual variability in measures of extinction and whether relationships between the measures were consistent with group differences. The final PCA used *n* = 51 rats (note that because of a technical issue, *n* = 1 young adult WT rat was removed). The Kaiser–Meyer–Olkin measure of sampling adequacy was 0.630, and Bartlett’s test of sphericity was significant (approximate χ^2^_(10)_ = 27.294, *p* = 0.002), indicating a correlation between loaded variables. Item communalities were moderate to high (range, 0.577–0.668) for all items except the I/O peak amplitude, which was low (0.431).

**Figure 6.**
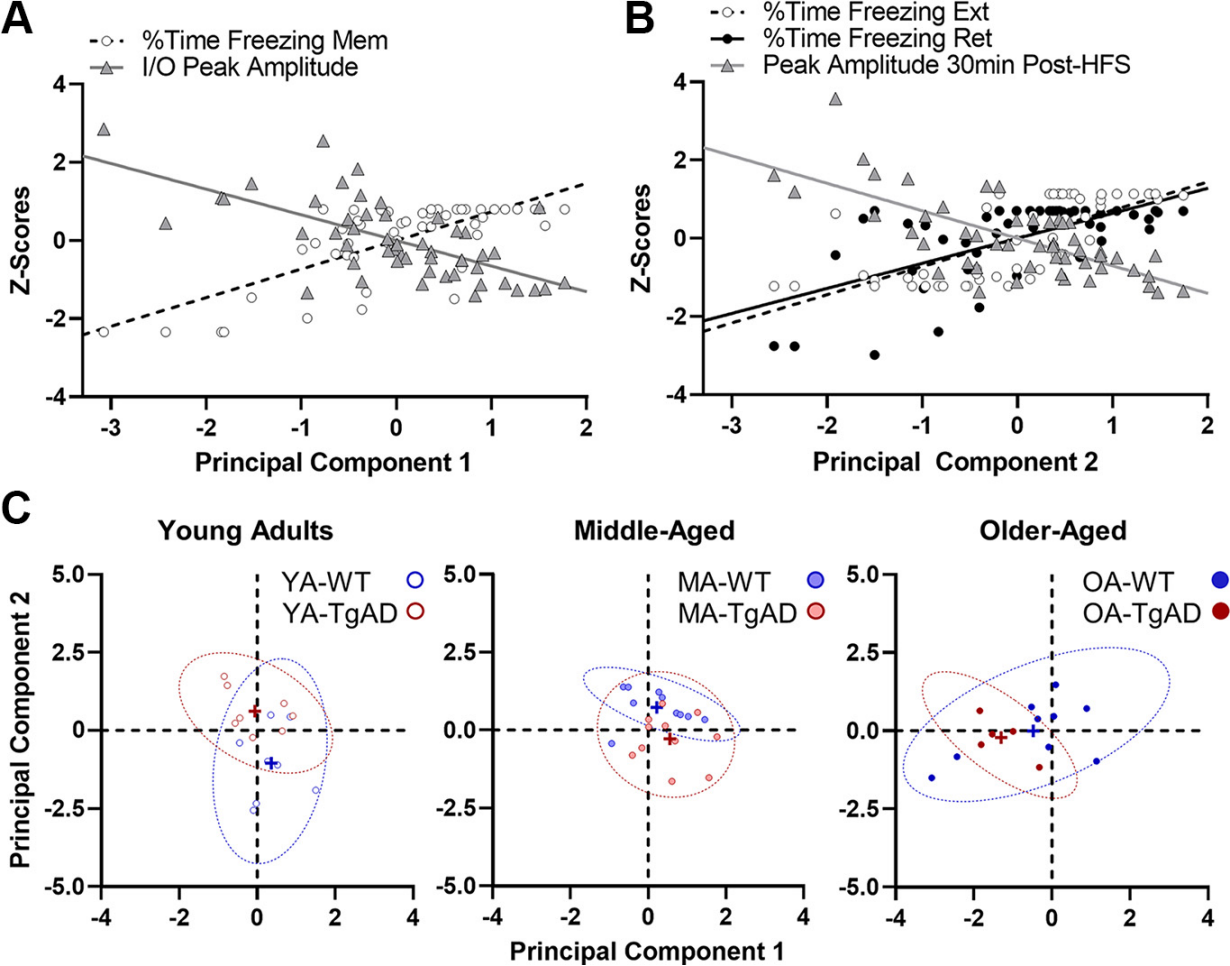
Principal component analysis on measures of CS extinction and BA synaptic function. ***A***, Significant correlations with component 1. In rats with large I/O peak amplitudes, there was also less freezing during the memory probe trial (Mem). ***B***, Significant correlations with component 2. In rats with impaired LTP, there was also impaired extinction and extinction memory retrieval. ***C***, Group differences in the contributions to each component resulted in unique clustering. Component 1 segregated rats by age such that all older-aged rats significantly clustered more negatively than young or middle-aged rats, indicating that the rats at the tail end of the correlations with component 1 displayed in ***A*** were older-aged rats. In contrast, component 2 segregated groups by age and genotype such that young adult WT rats clustered more negatively relative to their middle-aged and older-aged WT counterparts and their young TgAD counterparts. This confirmed that the rats at the negative tail of the correlations with component 2 displayed in ***B*** were young adult WT rats. Additionally, middle-aged TgAD rats also clustered more negatively than their young TgAD or middle-aged WT counterparts, indicating that the rats more positively adjacent to young WT rats in the correlations in ***B*** were middle-aged TgAD rats. While the middle-aged TgAD rats had a minor young-like phenotype, the more positive shift was likely because of the impairment in extinction memory retrieval. In ***C***, crosses represent group means, and dotted ellipses represent the 95% confidence intervals.

A model with two components (eigenvalues >1) explained 59.15% of the variance in the data. Component 1, which explained 38.18% of the variance, loaded positively with freezing during the CS memory probe trial (*r* = +0.731), but loaded negatively with the magnitude of peak amplitudes across all stimulation intensities (*r* = −0.656). These data indicate that rats with less fear memory expression during the CS memory probe (Mem) had a more hyperexcitable BA (Fig. 6*A*). In contrast, component 2, which explained 20.97% of the variance, loaded positively with freezing during extinction (*r* = +0.722) and extinction memory retrieval (*r* = +0.640), but loaded negatively with the magnitude of peak amplitudes at 30 min post-HFS (*r* = −0.704). These data indicate that rats with impaired extinction and extinction memory retrieval also had impaired BA LTP (Fig. 6*B*).

We then tested whether the explained variance segregated age and genotype groups uniquely by component. Figure 6*C* shows component 1 significantly segregated groups by age (*F*_(2,45)_ = 8.899, *p* < 0.001, η_p_^2^ = 0.283, 1-β = 0.963) such that older-aged rats clustered more negatively relative to young adult rats (−1.037, *p* = 0.003) and middle-aged rats (−1.277, *p* < 0.001), whereas genotype had no effect or did not interact with age (*F* values_(1–2,45)_ = 1.446–1.927, *p* values = 0.157–0.236). This indicated that older-aged rats with the highest I/O peak amplitudes also showed the lowest freezing during the Mem trial. In other words, older-aged rats clustered toward the negative tail of the correlations with component 1. Figure 6*C* also shows component 2 significantly segregated age groups by genotype (*F*_(2,45)_ = 11.444, *p* < 0.001, η_p_^2^ = 0.337, 1-β = 0.990) with no main effects (*F* values_(1–2,45)_ = 0.427–1.444, *p* values = 0.247–0.517). Specifically, young adult WT rats clustered more negatively relative to middle-aged rats (−1.776, *p* < 0.001) and older-aged WT rats (−1.042, *p* = 0.014) and their young adult TgAD counterparts (−1.664, *p* < 0.001). This indicated that young adult WT rats as a group showed the best fear extinction, extinction memory retrieval, and largest peak amplitudes at 30 min post-HFS. Indeed, the young adult WT rats clustered toward the negative tail of the correlations with component 2. Interestingly, middle-aged TgAD rats also clustered more negatively relative to young adult TgAD rats (−0.874, *p* = 0.031) and their middle-aged WT counterparts (−0.986, *p* = 0.010). Although they were adjacent to the young adult WT rats, the middle-aged TgAD rats did not occupy the same portion of the correlation that young adult WT rats occupied, as they were impaired in extinction memory retrieval despite having intact extinction and LTP. Together, these data suggest two conclusions. First, regardless of genotype, BA hyperexcitability in old age may impact the degree to which fear memory is expressed, though not necessarily fear memory per se. Second, BA LTP facilitates the degree to which fear memory extinction and extinction memory retrieval occur. Importantly, these results are consistent with the age × genotype interactions in the behavioral and electrophysiological measures noted above and further agree with the compensatory mechanisms observed in the middle-aged TgAD rats that do not extend into old age.

**Table 4:** Stimulus–response paired-pulse ratios

Intensity	Mean	SE	95% Confidence interval	
			Lower bound	Upper bound
20	0.036	0.016	0.004	0.067
25	0.067	0.024	0.019	0.116
30	0.103	0.025	0.053	0.153
35	0.141	0.029	0.083	0.200
40	0.121	0.024	0.073	0.168
45	0.099	0.024	0.050	0.148
50	0.077	0.025	0.028	0.127
55	0.034	0.022	−0.010	0.079
60	0.000	0.022	−0.045	0.044
65	−0.035	0.020	−0.076	0.006
70	−0.036	0.019	−0.074	0.003
75	−0.043	0.021	−0.085	0.000
80	−0.068	0.022	−0.111	−0.024
85	−0.051	0.020	−0.092	−0.011
90	−0.063	0.022	−0.108	−0.018
95	−0.093	0.025	−0.144	−0.042
100	−0.114	0.037	−0.188	−0.040

Mean and SE of the PPR for each stimulation intensity regardless of age and genotype.

## Discussion

In summary, nonpathologic aging and AD impair extinction memory and generalize contextual fear memory. While aging impairs LTP overall, it occurs later in TgAD rats, and the BA is hyperexcitable across the life span in TgAD rats but not in WT rats. Importantly, these findings are not explained by differences in shock perception, environmental habituation (Oler and Markus, 1998; Moyer and Brown, 2006; Luo et al., 2015; Zhan et al., 2018), or the prestimulus period during synaptic recordings.

### Fear memory acquisition in the presence of age and AD

Consistent with no genotypic difference in fear acquisition in young and middle-aged rats, a recent study from our laboratory reported no differences in contextual fear acquisition in a mixed cohort of young and middle-aged WT and TgAD rats given a series of uncued footshocks (Goodman et al., 2021). Aging, however, attenuated acquisition in middle-aged rats, spared acquisition in older-aged WT rats, and impaired acquisition in older-aged TgAD rats, consistent with studies showing aging attenuates, but does not grossly impair, acquisition (Villarreal et al., 2004; Kaczorowski et al., 2012). Given a larger number of trials or shorter delays between CS onset and US delivery, it is possible that all middle-aged rats and older-aged TgAD rats would show enhanced fear acquisition. Indeed, one study showed that acquisition-impaired rats can eventually acquire greater fear memory responses with overtraining (Maren, 1999), whereas another study showed long traces before a footshock impaired association between CS and US in older-aged rats (age range, 23–25 months; McEchron et al., 2004). Moreover, several studies have shown that aged rats acquire fear memory more robustly when trained with short delays between CS onset and US delivery (Villarreal et al., 2004; Moyer and Brown, 2006; Detert et al., 2008). Critically, our results in older-aged TgAD rats are consistent with impaired fear memory acquisition in Alzheimer’s patients (Hamann et al., 2002; Hoefer et al., 2008).

### The CS memory probe trial confirms spared consolidation

Although there were age-related and AD-related deficits in acute acquisition, rats at all ages showed increased fear memory expression during the memory probe trial, suggesting that there was a 24 h fear memory incubation period (Herry et al., 2008). The fact that acute acquisition deficits were overcome suggests some degree of intact fear memory consolidation and retrieval. The BA has two populations of glutamatergic neurons, known as “fear” and “extinction” neurons (Herry et al., 2008; Amano et al., 2011; Pare and Duvarci, 2012). One hypothesis for the emergence of memory in the older-aged TgAD rats is that hyperexcitable BA fear neurons facilitated consolidation and retrieval. Indeed, older-aged TgAD rats showed greater BA hyperexcitability. Importantly, the fear response displayed by each group was within an appropriate parametric space to detect CS extinction.

### Acute CS extinction in the presence of age and AD

Greater fear expression in young TgAD rats during the probe trial is consistent with enhanced fear to cued footshocks in AD mouse models (España et al., 2010). Furthermore, impaired extinction in young TgAD rats is consistent with deficits in 4.5-month-old APP^swe^/PS1^ΔE9^ mice (Bonardi et al., 2011) and TASTPM mice (Rattray et al., 2009). Fear extinction requires that retrieved memories be labile and conducive to inhibition and updating before reconsolidation (Kida, 2019). Moreover, BA extinction neurons may need to be disinhibited from upstream hyperexcitable fear neurons via an intermediary inhibitory circuit to permit extinction (Pare and Duvarci, 2012). Therefore, the hyperexcitability at BA synapses in young TgAD rats suggests an insurmountable drive on fear neurons, rendering the memory resistant to a labile state. Additionally, attenuated LTP in young TgAD rats suggests an inflexible circuit and further undermines the reinforcement of extinction. These data further support the evidence that emotional memory deficits are an early sign of AD (Hamann et al., 2002; Hoefer et al., 2008) and are consistent with known amygdala-related deficits in AD (Wright et al., 2007).

Though unexpected, enhanced extinction in middle-aged TgAD rats is consistent with a previous study reporting that 3×Tg-AD mice were better than WT mice during extinction (Pietropaolo et al., 2008). As detailed above, it is unlikely that diminished salience to the US explains spared extinction, though circuit-level compensation could offer one explanation. Despite hyperexcitability in the BA of middle-aged TgAD rats, synaptic strength and LTP mimic those in young adult WT rats. This young-WT-like phenotype may facilitate acute extinction encoding but not extinction memory. Though aging is not usually emphasized in rodent models of AD, morphologic and synaptic function changes occur in the absence of cell death in the BLA of middle-aged APP^swe^/PS1^ΔE9^ mice (Knafo et al., 2009), suggesting that middle age is accompanied by a local network restructuring before neuronal loss that facilitates an ephemeral compensation in extinction and BA function.

Compensation in middle-aged TgAD rats does not extend into older age. The persistence of BA hyperexcitability in middle-aged TgAD rats may set the stage for a vulnerable environment negatively impacted by aging. Unfortunately, there is a paucity of studies in rodent models of AD that also address age-related deficits. However, consistent with our findings, fear extinction is progressively worse in AD relative to patients with mild cognitive impairment (MCI) and healthy control subjects (Nasrouei et al., 2020), and AD patients show heightened amygdala responses to negative stimuli (Wright et al., 2007). Young adult and middle-aged TgAD rats may be displaying memory deficits akin to MCI, and by old age those deficits are compounded into clear impairments in emotional memory driven by underlying pathologic changes resulting in hyperactive and rigid BA synapses.

One strength of our study is addressing aging within the context of AD. Indeed, our results provide further evidence that nonpathologic aging is distinct from pathologic aging, as extinction and synaptic deficits in WT rats across the life span do not mirror those in TgAD rats. A second strength of our study is the inclusion of a middle-aged cohort to better define the trajectory of outcomes across the life span. Similarly, another study showed impaired extinction in middle-aged WT rats (Kaczorowski et al., 2012) and some degree of extinction in older-aged WT rats, albeit attenuated in magnitude (Moyer and Brown, 2006; Kaczorowski et al., 2012). However, Villarreal et al. (2004) reported impaired extinction in 22-month-old rats. While impaired LTP in the BA of middle-aged WT rats is novel, consistent with our own findings, older age is accompanied by impaired LTP (Zeng et al., 2012; Zhan et al., 2018). Our paired behavioral and electrophysiology results in older aged rats were not surprising given a wide body of literature showing older adults and rats depend on differential neural recruitment to achieve equal performance (Samanez-Larkin et al., 2011; Antonenko and Flöel, 2014; Lighthall et al., 2014; Tomás Pereira et al., 2015; Wang et al., 2015; Hernandez et al., 2019). Notably, these studies did not incorporate a middle-aged group to determine whether age differences are progressive, abrupt, or dynamic (i.e., whether phenotypic worsening occurs before compensation). As such, older-aged WT rats may require extra-amygdalar circuit recruitment during extinction, which is absent in middle-aged WT rats.

### Extinction memory is impaired in aging and AD

Aging and AD significantly impaired extinction memory retrieval. Only young adult WT rats retrieved extinction memory. Fear memories that undergo extinction are reconsolidated as new memories, while the original memory remains with less saliency (Bouton and King, 1983; Barad et al., 2006). However, in rats showing acute extinction, it is difficult to determine whether impaired retrieval was because of poor reconsolidation or an impairment in retrieval itself. Nonetheless, both mnemonic mechanisms are supported by the BLA, and the BA nucleus specifically (Maren, 2001; Kochli et al., 2015), and as such, retrieval in aging and AD may rely on an intact BA for extinction encoding and reconsolidation. Although more testing is necessary, our data suggest it is possible that synaptic compensation in middle-aged TgAD rats affords the encoding of acute extinction memory that does not appropriately reconsolidate, whereas additional circuit recruitment facilitates extinction encoding but not reconsolidation in older-aged WT rats. The associations between extinction retrieval and synaptic function in aged WT rats and all TgAD rats deviate from young WT, suggesting that any combination of synaptic deficits results in long-term extinction deficits. Although the BLA is a focus for fear memory encoding, consolidation, and storage, other neural circuitry may compensate when BLA deficits are present (Cahill et al., 2000; Maren, 2001; Kochli et al., 2015), particularly in aging (Hernandez et al., 2019).

### Context discrimination is impaired in aging and AD

To test contextual fear memory generalization, analyses focused on fear memory expression after contexts were associated with being either safe or unsafe. While the context renewal results suggest intact contextual discrimination in young adult and older-aged WT rats, older-aged WT rats showed greater fear responses in all context testing, thus suggesting a maladaptive response despite clear discrimination between contexts. Consistent with the current study, 23-month-old rats demonstrated comparable fear expression during contextual renewal testing relative to young adults (Oler and Markus, 1998). In all other groups, however, there were progressive increases in contextual fear memory expression, suggesting a maladaptive response similarly observed in neuropsychiatric disorders like post-traumatic stress disorder (Garfinkel et al., 2014; Huckleberry et al., 2016). Although the hippocampus is critical in contextual fear memory consolidation (Phillips and LeDoux, 1992), there is evidence of hippocampal-independent mechanisms of consolidation (Wiltgen et al., 2006; Kochli et al., 2015). The context deficits in TgAD rats are similar throughout all stages of life, and it is reasonable to suggest that the contribution to extinction impairments and contextual generalization is shared between persistent BA hyperactivity and known hippocampal deficits in this rat model (Smith and McMahon, 2018; Goodman et al., 2021). In nonpathologic aging, contextual fear memory deficits may also be explained by BA and hippocampal dysfunction, given the wide body of literature showing age-related impairments to hippocampal function (Comery et al., 2005; Ohno, 2009; Zeng et al., 2012; Fjell et al., 2014; Zhan et al., 2018; Burke and Foster, 2019).

### Conclusions and future studies

These results emphasize the unique trajectories that aging takes in the presence and absence of disease. As AD risk significantly increases with age, and potential compensatory mechanisms may confound interpretations of data that do not account for age, more studies would benefit from incorporating age as a factor when using rodent models of AD. While we show clear synaptic deficits in the BA of aged and TgAD rats that also have extinction memory deficits, we cannot determine the underlying cause of BA synaptic deficits. Indeed, future studies will seek to determine the degree to which inflammation, amyloid, and tau pathology play a role in BA synaptic dysfunction and extinction memory deficits.

## Synthesis

Reviewing Editor: Karyn Frick, University of Wisconsin-Milwaukee Decisions are customarily a result of the Reviewing Editor and the peer reviewers coming together and discussing their recommendations until a consensus is reached. When revisions are invited, a fact-based synthesis statement explaining their decision and outlining what is needed to prepare a revision will be listed below. The following reviewer(s) agreed to reveal their identity: NONE. Note: If this manuscript was transferred from JNeurosci and a decision was made to accept the manuscript without peer review, a brief statement to this effect will instead be what is listed below.

Thank you for transferring your manuscript to eNeuro. I have reviewed the revisions to your manuscript and determined that they adequately addressed the reviewer’s concerns. Thus, I have decided to accept it as is rather than sending it out for re-review.
